# 5-Hydroxymethylcytosine Signatures in Circulating Cell-Free DNA as Early Warning Biomarkers for COVID-19 Progression and Myocardial Injury

**DOI:** 10.3389/fcell.2021.781267

**Published:** 2022-01-06

**Authors:** Hang-yu Chen, Xiao-xiao Li, Chao Li, Hai-chuan Zhu, Hong-yan Hou, Bo Zhang, Li-ming Cheng, Hui Hu, Zhong-xin Lu, Jia-xing Liu, Ze-ruo Yang, Lei Zhang, Nuo Xu, Long Chen, Chuan He, Chao-ran Dong, Qing-gang Ge, Jian Lin

**Affiliations:** ^1^ Department of Pharmacy and Department of Intensive Care Unit, Peking University Third Hospital, Beijing, China; ^2^ Institute of Materia Medica, Chinese Academy of Medical Sciences and Peking Union Medical College, Beijing, China; ^3^ Institute of Biology and Medicine, College of Life and Health Sciences, Wuhan University of Science and Technology, Hubei, China; ^4^ Department of Laboratory Medicine, Tongji Hospital, Tongji Medical College, Huazhong University of Science and Technology, Wuhan, China; ^5^ Department of Medical Laboratory, the Central Hospital of Wuhan, Tongji Medical College, Huazhong University of Science and Technology, Wuhan, China; ^6^ Yang Sheng Tang Natural Medicine Research Institute, Hangzhou, China; ^7^ Synthetic and Functional Biomolecules Center, Beijing National Laboratory for Molecular Sciences, Key Laboratory of Bioorganic Chemistry and Molecular Engineering of Ministry of Education, College of Chemistry and Molecular Engineering, Innovation Center for Genomics, Peking University, Beijing, China; ^8^ Department of Chemistry, Department of Biochemistry and Molecular Biology, Howard Hughes Medical Institute, The University of Chicago, Chicago, IL, United States

**Keywords:** COVID-19, 5hmC, myocardial injury, machine learning, PDE4D

## Abstract

**Background:** The symptoms of coronavirus disease 2019 (COVID-19) range from moderate to critical conditions, leading to death in some patients, and the early warning indicators of the COVID-19 progression and the occurrence of its serious complications such as myocardial injury are limited.

**Methods:** We carried out a multi-center, prospective cohort study in three hospitals in Wuhan. Genome-wide 5-hydroxymethylcytosine (5hmC) profiles in plasma cell-free DNA (cfDNA) was used to identify risk factors for COVID-19 pneumonia and develop a machine learning model using samples from 53 healthy volunteers, 66 patients with moderate COVID-19, 99 patients with severe COVID-19, and 38 patients with critical COVID-19.

**Results:** Our warning model demonstrated that an area under the curve (AUC) for 5hmC warning moderate patients developed into severe status was 0.81 (95% CI 0.77–0.85) and for severe patients developed into critical status was 0.92 (95% CI 0.89–0.96). We further built a warning model on patients with and without myocardial injury with the AUC of 0.89 (95% CI 0.84–0.95).

**Conclusion:** This is the first study showing the utility of 5hmC as an accurate early warning marker for disease progression and myocardial injury in patients with COVID-19. Our results show that phosphodiesterase 4D and ten-eleven translocation 2 may be important markers in the progression of COVID-19 disease.

## Introduction

The pandemic of coronavirus disease 2019 (COVID-19), caused by severe acute respiratory syndrome coronavirus 2 (SARS-CoV-2) infection, has become a global public health crisis (Pollard et al., 2020). According to the World Health Organization (WHO) latest numbers on July 21, 2021, over 192.8 million confirmed cases of COVID-19 and more than 4.0 million deaths worldwide. The clinical spectrum of COVID-19 pneumonia ranges from asymptomatic infection to critically ill cases (McArthur et al., 2020). Critical patients with a higher mortality rate suffered from organ failure, including cerebrovascular accident (CVA), myocardial injury (MI), and thrombotic events (Wang et al., 2020). MI has been the most reported cardiovascular complication with a significant risk of in-hospital mortality rate (51.2%) compared with those without MI (4.5%) (Bonow et al., 2020). These findings suggest that early identification of patients with COVID-19 at risk of critical illness could improve their outcomes. Recently, several studies demonstrated that the higher levels of inflammatory markers such as C-reactive protein (CRP) (Wang, 2020), ferritin (Lin et al., 2020), D-dimer (Zhang et al., 2020), high neutrophil-to-lymphocyte ratio (NLR) (Kong et al., 2020), and blood proteomic and metabolomic biomarkers (Wu D. et al., 2020; Shen et al., 2020) could be used to distinguish between moderate and severe cases. Unfortunately, so far there are no reliable indicators available to warn the COVID-19 progression and the occurrence of serious complications such as MI.

5-Hydroxymethylcytosine (5hmC) is an abundant epigenetic marker associated with gene expression and involves a wide range of biological processes ranging from development to pathogenesis (Han et al., 2016). It is derived from 5mC by ten-eleven translocation (TET) protein family and displays a tissue-specific mass distribution (Tan and Shi, 2012). Our laboratory and others have demonstrated that the 5hmC signatures from cell-free DNA (cfDNA) could serve as epigenetic biomarkers for several human diseases such as cancer, neurodegenerative disorders, and coronary heart disease (Szulwach et al., 2011; Li et al., 2017; Dong et al., 2020). These characteristics indicate that 5hmC may have potential value in COVID-19 warning and discovery of target therapy.

In this study, we performed 5hmC-Seal, a sensitive chemical labeling-based sequencing technology (Song et al., 2011; Dong et al., 2020) that allows rapid and reliable sequencing of whole-genome 5hmC in cfDNA from plasma of 256 patients with (*n* = 203) and without (*n* = 53) COVID-19 diagnosis. We found 5hmC characteristics detected in cfDNA could be used as early warning markers for the disease progression and MI of COVID-19.

## Materials and Methods

### Data and Sample Source

We carried out a multi-center, prospective cohort study in three hospitals in Wuhan. From March to April 2020, we consecutively enrolled 203 patients aged at least 18 years and diagnosed with COVID-19 within 48 h after their hospitalization. Patients were classified into three groups according to the disease severity defined by the National Health Commission of the People’s Republic of China (Table 1) (National Health Commission, 2020). Five-milliliter discarded plasma samples were collected from each patient as they were entering into the study cohort, and the results of clinical tests nearby were recorded. The plasma cfDNA was extracted using the Quick-cfDNA Serum and Plasma Kit (ZYMO) and then stored at −80°C. Each patient was prospectively followed up until hospital discharge or death. Complications during the first 28-day follow-up were evaluated and recorded: 1) MI, based on symptoms (if described), electrocardiogram/echocardiography (if any), and troponin I (TnI) (≥0.4 ng/ml) (Dong et al., 2020); 2) gastrointestinal injury (GS), defined as occurrence of gastrointestinal haemorrhage, gastroparesis, and severe/acute pancreatitis; 3) sepsis-induced coagulopathy (SIC), defined as blood platelet <150*10^9^/L. The occurrence of all-cause death during the whole follow-up was confirmed by home page and final discussion in medical records. The Ethical Review Board of the Peking University Third Hospital approved the study protocol in March 2020 (IRB00006761-M2020083). On the basis of the consideration of discarded samples used, together with a complete set of information security system established, the informed consent of participants was exempted.

**TABLE 1:** Statistical characteristics of baseline indicators in patients with COVID-19.

	Total (*n* = 203)	Moderate (*n* = 66)	Severe (*n* = 99)	Critical (*n* = 38)	*p*-value
Age, years	65 (54–73)	58 (37–67)	67 (57–77)	67 (56–73)	***
					—
					§
≥65 years	74 (68–81)	72 (67–83)	77 (69–81)	72 (68–74)	—
Gender, female/male	98/105	40/26	43/56	15/23	—
Obesity (BMI ≥30)	4 of (22)	0 of 0	3 of 21	1 of 1	
Hypertension	84	16	47	21	
Coronary heart disease	18	2	6	10	
Heart failure	12	1	4	7	
Chronic liver disease	13	0	4	9	
Immunodeficiency	9	1	2	6	
Stroke history	12	2	3	7	
Diabetes	47	7	16	24	
Asthma, moderate severe	3	0	1	2	
COPD	14	0	6	8	
CKD	15	1	2	12	
Cancer	8	0	1	7	
Smoke history, naïve/ex-smoker/smoker	119/14/18	18/2/3	75/9/6	26/3/9	

a *n* (§), median (p25-p75), mean ± SD.

b *p*-value: Moderate-Severe: *, Severe-Critical: #, Moderate-Critical: §.

c *p < 0.05, **p < 0.01, and ***p < 0.001.

COPD, chronic obstructive pulmonary disease; CKD, chronic kidney disease.

### Study Design

**FIGURE 1:**
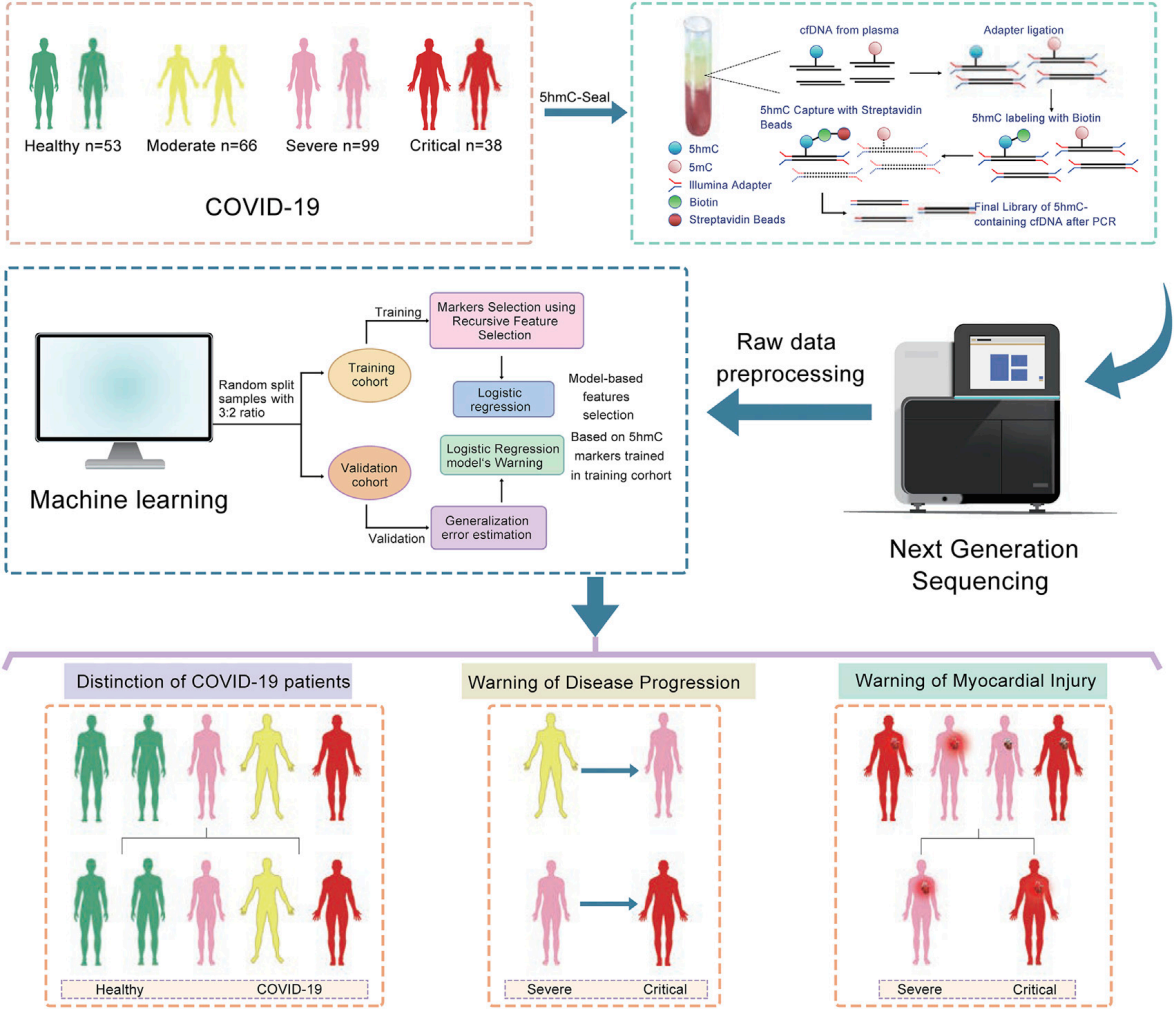
Overview of study design. A total of 203 cfDNA samples were collected at the time of diagnosis from patients with COVID-19. 5hmC libraries for all samples were constructed with high-efficiency hmC-Seal technology. cfDNA is ligated with Illumina adapter and labeled with biotin on 5hmC for pull-down with streptavidin beads. The final library is completed by direct PCR from streptavidin beads. Next, high-throughput sequencing was performed on the NextSeq 500 platform. A logistic regression model was trained by the training cohort was used to warn COVID-19 progression and myocardial injury in the validation cohort.

We performed a prospective cohort study using 5hmC markers to distinguish patients with COVID-19 from healthy people and warn the disease progression and MI. The 256 samples were divided into four groups: healthy people [*n* = 53, aged 35 (IQR 29–40) years], moderate patients (*n* = 66), severe patients (*n* = 99), and critical patients (*n* = 38). 5hmC libraries for all samples were constructed with high-efficiency 5hmC-Seal technology, as previously described (Song et al., 2011). All 5hmC libraries were sequenced using Illumina Next500. Meanwhile, in data processing, we split patients with COVID-19 into a training cohort and a validation cohort. The objective of the first part of the study was to screen candidate genes with differential 5hmC modifications in these four groups from the training cohort. The objective of the second part of the study was to warn disease progression and MI using the model developed in the first part, in the validation cohort (Figure 1).

### 5hmC Library Construction, Sequencing, and Mapping

Briefly, because of the highly sensitive nature of the chemical labeling method, the input cfDNA can be as low as 1–10 ng. Paired-end 39–base pair (bp) high-throughput sequencing was performed on the NextSeq 500 platform. FASTQC (version 0.11.5) was used to assess the sequence quality. Raw reads were aligned to the human genome (version hg19) with bowtie2 (version 2.2.9) (Langmead and Salzberg, 2012) and further filtered with SAMtools (version 1.3.1) (Li et al., 2009) to retain unique non-duplicate matches to the genome. Pair-end reads were extended and converted into bedGraph format normalized to the total number of aligned reads using BEDTools (version 2.19.1) (Quinlan, 2014) and then converted to bigwig format using bedGraphToBigWig from the UCSC Genome Browser for visualization in the Integrated Genomics Viewer. Potential hMRs were identified using MACS (version 1.4.2), and the parameters used were macs 14-p 1e-3-f BAM-g hs (Consortium et al., 2007). Peak calls were merged using BEDTools merge, and only those peak regions that appeared in more than 10 samples and less than 1,000 bp were retained. Blacklisted genomic regions that tend to show artifact signals, according to ENCODE, were also filtered.

### Feature Selection, Model Training, and Validation

Patients with COVID-19 were randomly divided into training and validation cohorts with a 3:2 ratio; using train_test_split in scikit-learn (version 0.22.1) package in *Python* (version 3.6.10), the logistic regression CV (LR) model was chosen to establish warning models. In the training cohort, we identified differentially 5hMc-enriched regions (DhMRs) using DESeq2 package (version 1.30.0) in R (version 3.5.0), with the filtering threshold (*p*-value< 0.01 and |log2FoldChange| ≥ 0.5). To avoid overfitting, five rounds of 10-fold cross-validation was performed. The details are as follows: The training cohort was randomly divided into fivefolds, four of which were selected as the training subset, and the remaining one was the test subset. Then, we performed 100 repeats to further filtered using the recursive feature elimination algorithm (RFECV) in scikit-learn [parameters used: estimator = LogisticRegressionCV (class_weight = “balanced”, cv = 2, max_iter = 1,000), scoring = “accuracy”]. Meanwhile, 10-fold cross-validation was repeated 100 times in each round, and the final markers observed in at least three rounds were used to build the final warning model in the training cohort. Next, we trained the logistic regression CV model (LR) with the features selected from DhMRs (parameter used: maxiter = 100, method = “lbfgs”). Finally, the trained LR model was used to warn the progression and MI for patients with COVID-19 in the validation cohort. Receiver operating characteristic (ROC) analysis was used to evaluate model performance.

### Clinical Indicators Prediction Model Construction

For clinical data, continuous variables are presented as mean (SD), and categorical variables are presented as count (percentages). To understand the relationship between categorical/continuous variables and treatment outcome, Kruskal–Wallis test by ranks and χ2 test were used, respectively. A two-sided *p*-value of < 0.05 was considered to be statistically significant. The warning power of clinical data was estimated by generalized linear model function in R-base and pROC package (version 1.15.3) in R (version 3.6.2).

### Exploring Functional Relevance of the 5hmC Markers

We used the ChIPseeker R Package (version 1.20.0) (Yu et al., 2015) to annotate the DhMRs, and the genes closest to the marker regions were used for the following functional analyses. The Gene Ontology (GO) enrichment analysis (Biological Process) was done by the ClueGO (version 2.5.5) and CluePedia (version 1.5.5) plug-in from Cytoscape software (version 3.7.2). We used the following parameters: medium network specificity, Bonferroni step down pV correction, and two-sided hypergeometric test.

### GEO Datasets

For published RNA sequencing (RNA-seq) dataset, GSE150728 (Wilk et al., 2020) and GSE151879 (Chen et al., 2020), we downloaded the normalized expression values directly from Gene Expression Omnibus (GEO) database.

### Statistical Analysis

Statistical analysis in Table 1 was conducted in GraphPad Prism 5. We used two-tailed *t*-tests (paired or unpaired depending on the experiments) for normally distributed data. We used the percentile method to calculate 95% CIs, and *p*-value < 0.05 was considered statistically significant.

## Results

### Sample Collected and Clinical Sample Characteristic

Table 1 shows the baseline characteristics of the patients. Of the 203 patients with COVID-19 (105 males and 98 females, the median age was 65 years), 66 patients were diagnosed with moderate symptoms, 99 patients with severe symptoms, and 38 patients with critical symptoms. Those severe to critical ones had a heavy burden of comorbidities such as hypertension, diabetes, and coronary heart disease. The levels of lymphocyte count (1.1×10^9^/L) and neutrophil count (3.62×10^9^/L) at the time of hospital admission were in the lower limit. In this study, compared with patients with moderate COVID-19, those severe to critical patients showed elder age, active cellular immunity (lower lymphocyte but higher CD3^+^CD19^−^level), and increased inflammatory response [higher neutrophil and interleukin-6 (IL-6) level)] at the baseline (Table 2). In addition, there were more likely to have poor outcomes presented as more extended mechanical ventilation, more days of hospital, stay, and even death. During the first 28-day follow-up, MI, SIC, and GS occurred in 40, 10, and 9 patients, respectively. A total of 14 patients died in the hospital, with the median time of 28 (8, 32) days from entering into cohort to death occurring.

**TABLE 2:** Statistical characteristics of clinical indicators in patients with COVID-19.

	Total (*n* = 203)	Moderate (*n* = 66)	Severe (*n* = 99)	Critical (*n* = 38)	*p*-value
NEUT#, ×10^9^/L	3.62 (3.05–6.95)	3.425 (2.83–5.41)	4.62 (3.01–6.48)	8.05 (4.79–11.86)	*
					###
					§§§
LYMPH#, ×10^9^/L	1.10 (0.57–1.51)	1.44 (1.14–1.83)	0.81 (0.53–1.35)	0.69 (0.37–1.22)	***
					§§§
PLT, ×10^9^/L	194 (146.25–247.5)	202.5 (169–254.75)	181 (141–239.25)	194.5 (136.25–260)	
(CD3^+^CD19^−^) #,/ul	283 (77.19–708)	73.24 (67.12–84.75)	478.5 (222.25–901.5)	403.5 (192.75–597.75)	***
					§§§
(CD3^+^CD4^+^) #	41.8 (33.41–48.44)	39.04 (33.12–47.53)	46.84 (46.12–48.69)	44.19 (34.14–49.89)	
(CD3^+^CD8^+^) #	23.89 (18.91–30.59)	24.8 (19.99–32.58)	19.34 (13.45–24.27)	21.34 (17.85–29.08)	
IL-6, pg/ml	9.44 (2.26–28.7)	2.92 (1.50–8.59)	11.99 (3.18–27.07)	29.54 (15.09–56.56)	**
					§§
TnI, pg/ml	0.02 (0.01–2.5)	0.005 (0.0017–0.01)	0.03 (0.01–7.60)	0.03 (0.012–0.065)	***
					§§§
INR	1.02 (0.97–1.12)	0.99 (0.95–1.05)	1.02 (0.97–1.12)	1.12 (0.125–1.215)	*
					##
					§§§
Mechanical ventilation	45	0	25	20	
If yes, mechanical ventilation, hours	494 (392–696)	NA	456 (254–684)	504 (360–696)	§§§
Hospital length of stay, days	26 (17–36)	20.5 (11–29)	31 (21–41)	26.5 (22–39)	***
					§§§
Survival/non-survival	184/15	66/0	90/8	28/7	

### The Landscape of 5hmC Profile Between the Healthy Sample and Patients with COVID-19

**FIGURE 2:**
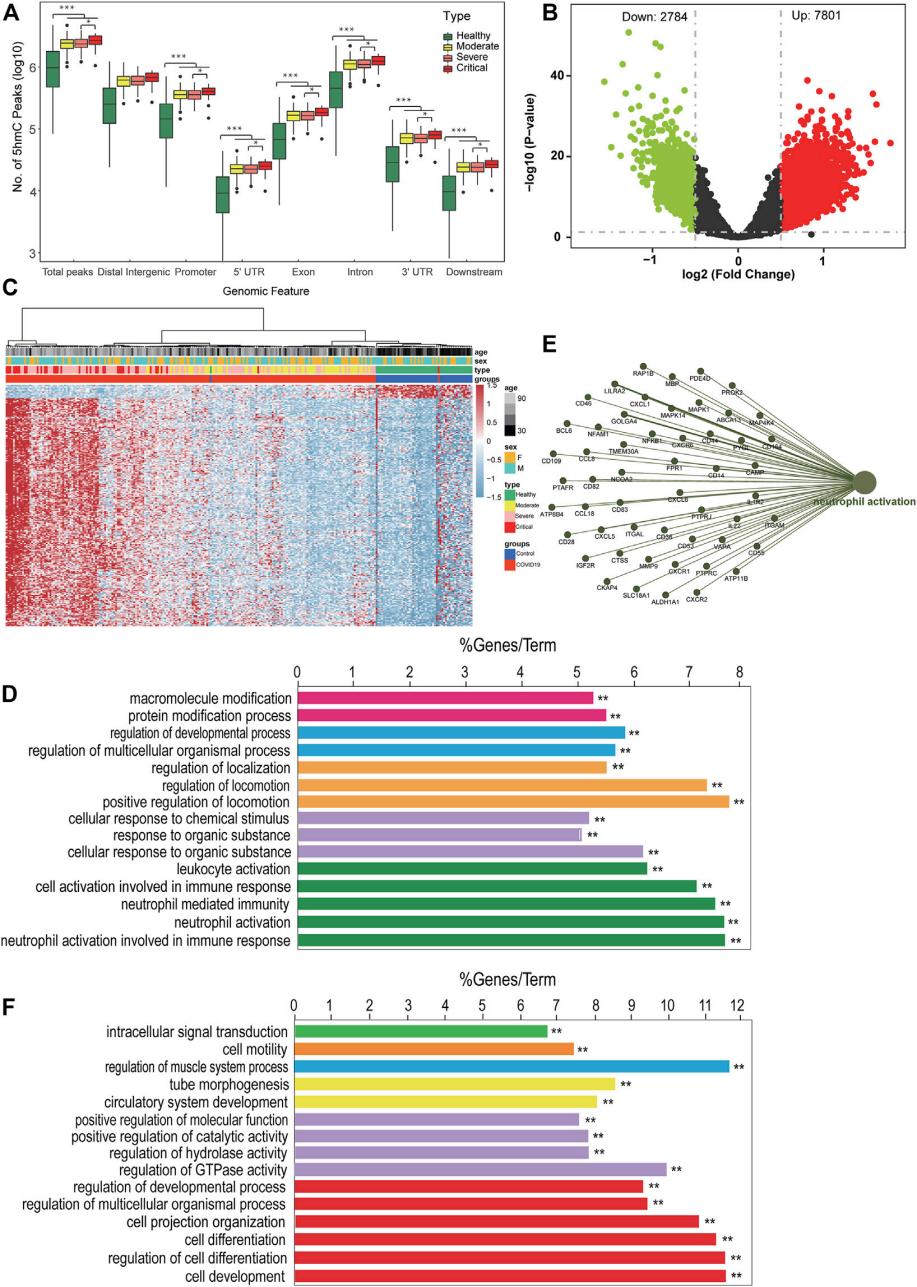
The landscape of 5hmC in circulating cell-free DNA of patients with COVID-19. **(A)** Genome-wide 5hmC distribution in different genomic features grouped by patients with COVID-19 (**p < 0.01). **(B)** Volcano plot (patients with COVID-19 vs. healthy control). Significantly altered hMRs [abs (log2FoldChange) ≥0.5; *p*-value <0.01] are highlighted in red (up) or green (down) using the COVID-19 groups as the reference. Black dots represent the hMRs that are not differences. **(C)** Heatmap of 203 patients with COVID-19 and 53 healthy control based on top 200 DhMRs (|log2FoldChange| ≥ 0.5 and *p* < 0.01). **(D–F)** GO enrichment analysis and function exploration of 5hmC markers using Cytoscape software (*p* < 0.01). GO enrichment with 5hmC increase **(D)** or decrease **(F)** in patients with COVID-19. **(E)** GO enrichment and Gene-Concept Network. hMRs, 5 hMc-enriched regions; DhMRs, differentially 5hMc-enriched regions.

According to the clinical presentation, the patients with COVID-19 were classified into three disease groups (Figure 1; Table 1). First, we perform quality control (QC) analysis for 5hmC-Seal data in four groups and each sample such as the unique mapping rate and number of unique reads (Supplementary Figure S1A-F, Supplementary Table S1). Then, we identified the 5hmC-enriched peaks among the four groups and found that the groups of patients with COVID-19 enriched more peaks than the healthy control group. This was more manifest in the critical group, which showed the highest 5-hmC in different genomic characteristic regions, such as promoters and exon (Figure 2A). In addition, we found that the groups of patients with COVID-19 have more peaks enriched in the enhancers (Supplementary Figure S1). Next, we conducted differential analysis (|log2FoldChange| ≥ 0.5, *p* < 0.01) and observed 10,585 DhMRs (differentially 5hmC enriched regions), including upregulated (*n* = 7,801) and downregulated (*n* = 2,784) regions in the patients with COVID-19 compared with the healthy group (Figure 2B, Supplementary Table S2). We clustered the top 200 DhMRs (190 up and 10 down) detected by hierarchical clustering method. The results showed that the COVID-19 groups were well separated from the healthy people group. Meanwhile, moderate, severe, and critical groups tended to differentiate from each other (Figure 2C).

Next, we did GO biological pathway analysis to explore the function pathway of signature 5hmC genes in patients with COVID-19. The result showed that genes with upregulated 5hmC signal were enriched in neutrophil cells mediated immune response pathway, such as neutrophil activation (Figure 2D); meanwhile, the hubs of the GO functional interaction networks showed that these 5hmC-associated differential genes (*n* = 52), including phosphodiesterase 4D (PDE4D), CD14 molecule (CD14), and mitogen-activated protein kinase kinase kinase 4 (MAP4K4) participated in regulating neutrophil activation pathway (Figure 2E). The pathway enrichment in the patients with COVID-19 was consistent with the previous studies, which reported the higher level of neutrophil-to-lymphocyte is associated with severe COVID-19 (Kong et al., 2020). Furthermore, the downregulated gene enriched pathways included cell development signaling pathways (Figure 2F). Strikingly, consistent with our findings, 5hmC-enriched genes involved with immune response signaling pathways had higher mRNA expression levels in neutrophils of patients with COVID-19 from the small conditional RNA-seq dataset (GSE150728); see Supplementary Figure S2A. In addition, CD14 (Supplementary Figure S2B) and MAP4K4 (Supplementary Figure S2C) were highly enriched in hydroxymethylation for patients with COVID-19 (*p* = 0.00033 and 0.044), and the levels of hydroxymethylation increased gradually in groups moderate, severe, and critical patients and MI. All these results suggest that differentially regulated 5hmC modified genes may have the potential to distinguish patients with COVID-19 from healthy people, and a unique combination of 5hmC modified genes would warn the disease progress.

### 5hmC as Early Warning Biomarkers for COVID-19 Progression

**FIGURE 3:**
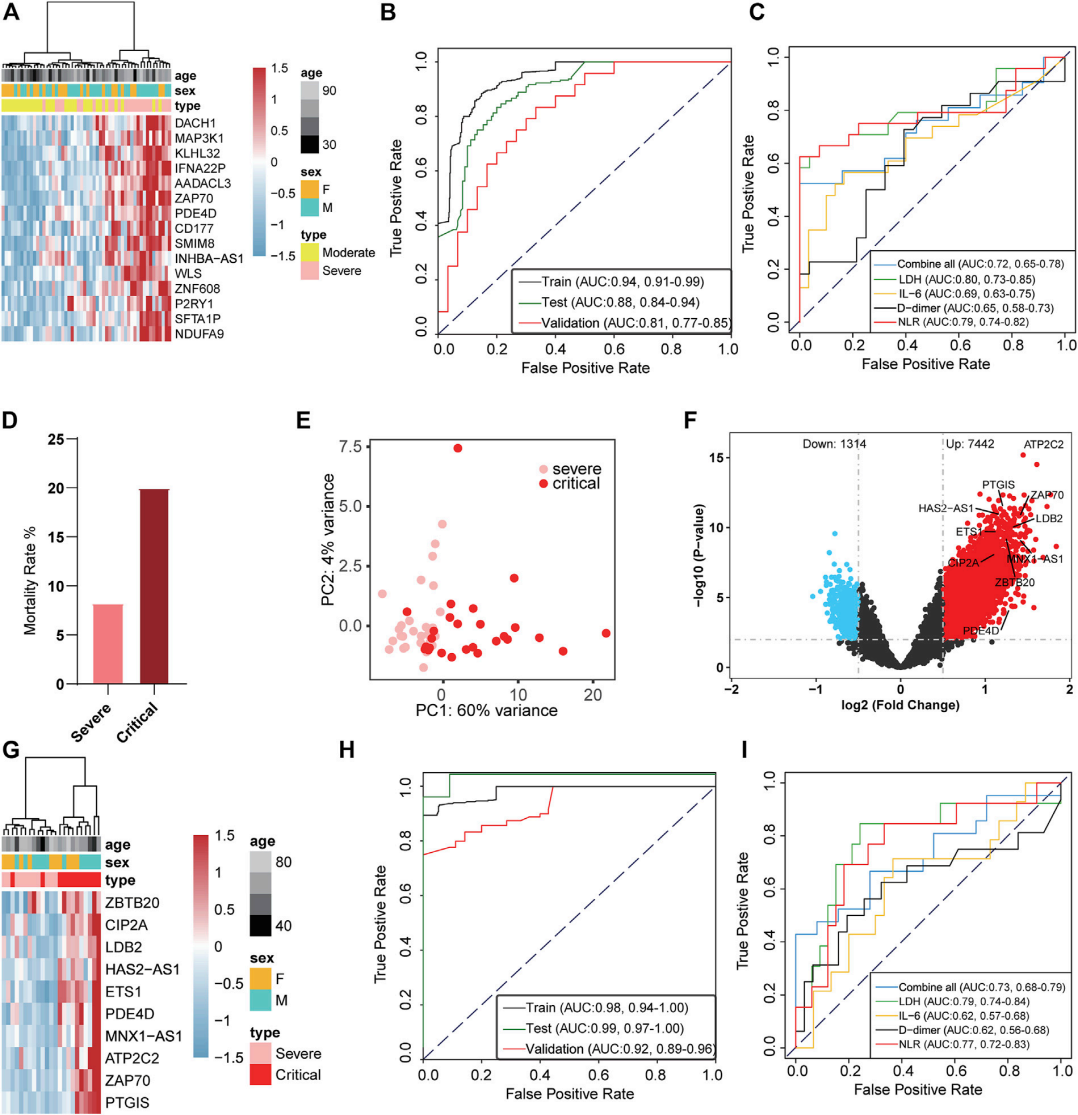
5-Hydroxymethylcytosine signatures in circulating cell-free DNA as a novel early warning biomarker for COVID-19 progression. **(A)** Heatmap of validation cohort based on 15 DhMRs-associated genes selected in the warning model. **(B)** Receiver operating characteristic (ROC) curve of the warning model with DhMRs in training and validation cohorts for COVID-19 progression. **(C)** ROC curve of the prediction model with clinical indicators in patients with COVID-19. **(D)** Mortality ratio in severe patients and critical patients. **(E)** Principal component analysis plot of normalized 5hmC reads from 27 severe patients and 26 critical patients. **(F)** Volcano plot (critical patients vs. severe patients). Significantly altered hMRs [abs (log2FoldChange) ≥ 0.5; *p*-value < 0.01] are highlighted in red (up) or blue (down) using the critical patients group as the reference (*n* = 8,756). Black dots represent the hMRs that are not differences. **(G)** Heatmap of DhMRs-associated genes selected for use in the warning model in the validation cohort. **(H)** ROC curve of the warning model with DhMRs in training and validation cohorts for COVID-19 progression. **(I)** ROC curve of the warning model with clinical indicators in COVID-19 progression. hMRs, 5hMc-enriched regions; DhMRs, differentially 5hMc-enriched regions.

We further analyzed whether 5hmC characteristics detected in cfDNA could be used as early biomarkers for COVID-19 progression. First, we investigated whether the candidate DhMRs were associated with the severity of the disease. A total of 132 patients with COVID-19 (66 with moderate and 66 with severe) were randomly divided into training (*n* = 78) and validation cohorts (*n* = 54). Using the RFECV based on the logistic regression CV estimator, we reduced the number of DhMRs (15 DhMRs, |log2FoldChange| ≥ 0.5, *p* < 0.01, Supplementary Table S3) in the training cohort, which achieved the best cross-validation score. We found the 15 DhMRs (Supplementary Table S9), selected by the LR model in the training cohort, that could distinguish severe patients from moderate patients in the training (Supplementary Figure S3A) and validation cohorts (Figure 3A). Fifteen DhMRs could effectively warn moderate patients and severe patients in the training cohort [area under the curve (AUC) = 0.94, 95% CI: 0.91–0.99] and the validation cohort (AUC = 0.81, 95% CI: 0.77–0.85); see Figure 3B. Recent studies demonstrated that uncontrolled inflammation contributes to disease severity in COVID-19 (Wu C. et al., 2020). By developing and validating our model, we also confirmed that certain inflammatory markers such as IL-6, D-dimer, NLR, and lactate dehydrogenase (LDH) could be used as predictors of COVID-19 disease severity. Notably, the combination of IL-6, D-dimer, NLR, and LDH as a warning indicator of disease progression achieved an AUC of 0.72 (95% CI: 0.65–0.78), which is lower than the 5 hmC indicators (Figure 3C).

A total of 18.7% (38 cases) of patients with COVID-19 rapidly developed a critical illness and had a higher mortality rate than severe illness (Figure 3D). Therefore, the warning of patients who become critically ill has clear significance for the early treatment of COVID-19. To investigate whether the candidate DhMRs were associated with critical patients, we randomly separated 38 severe patients and 38 critical patients into training (*n* = 53) and validation cohorts (*n* = 23). Then, we examined the difference in 5hmC regions between severe and critical patients in the training cohort. Principal component analysis (PCA) based on top variance genes showed that severe patients could separate from the critical patients based on the 5hmC patterns (Figure 3E). Meanwhile, we conducted differential analysis (|log2FoldChange| ≥ 0.5, *p* < 0.01) and observed 8,756 DhMRs, including upregulated (*n* = 7,442) and downregulated (*n* = 1,314) regions in severe compared to critical patients (Figure 3F and Supplementary Table S4). Using the RFECV based on the logistic regression CV estimator, we further reduced the number of top 200 DhMRs from the training cohort to 10 DhMRs (Supplementary Table S10), which achieved the best cross-validation score. In the validation cohort, 10 DhMRs differentiated severe patients from critical patients in the training (Supplementary Figure S3D) and validation cohorts (Figure 3G). The AUC value of this model for warning severe patients and critical patients was 0.92 (95% CI: 0.89 to 0.96, Figure 3H), which was much higher than the clinical indicators showed, such as LDH (AUC = 0.79, 95% CI: 0.74–0.84), IL-6 (AUC = 0.62, 95% CI: 0.57–0.68), D-dimer (AUC = 0.62, 95% CI: 0.56–0.68), and NLR (AUC = 0.77, 95% CI: 0.72–0.83); see Figure 3I. These results implied that the 5hmC-based biomarkers of circulating cfDNA were highly indicating of COVID-19 progression. Gene functional enrichment analysis showed that upregulated 5hmC modified genes in the severe and critical patients were mainly enriched in neutrophil degranulation, neutrophil-mediated immunity, and neutrophil activation involved in immune response (see Supplementary Figures S3B and S3C), which are associated with development and progression of acute respiratory distress syndrome (ARDS) (Wu C. et al., 2020). Interestingly, PDE4D was one of the warning biomarkers that might indicate moderate to severe illness and severe to critical illness and mediate cell chemotaxis signaling pathways to affect the neutrophil-related immune system in critical patients (Supplementary Figure S3E).

### 5hmC as Warning Biomarkers for Myocardial Injury

**FIGURE 4:**
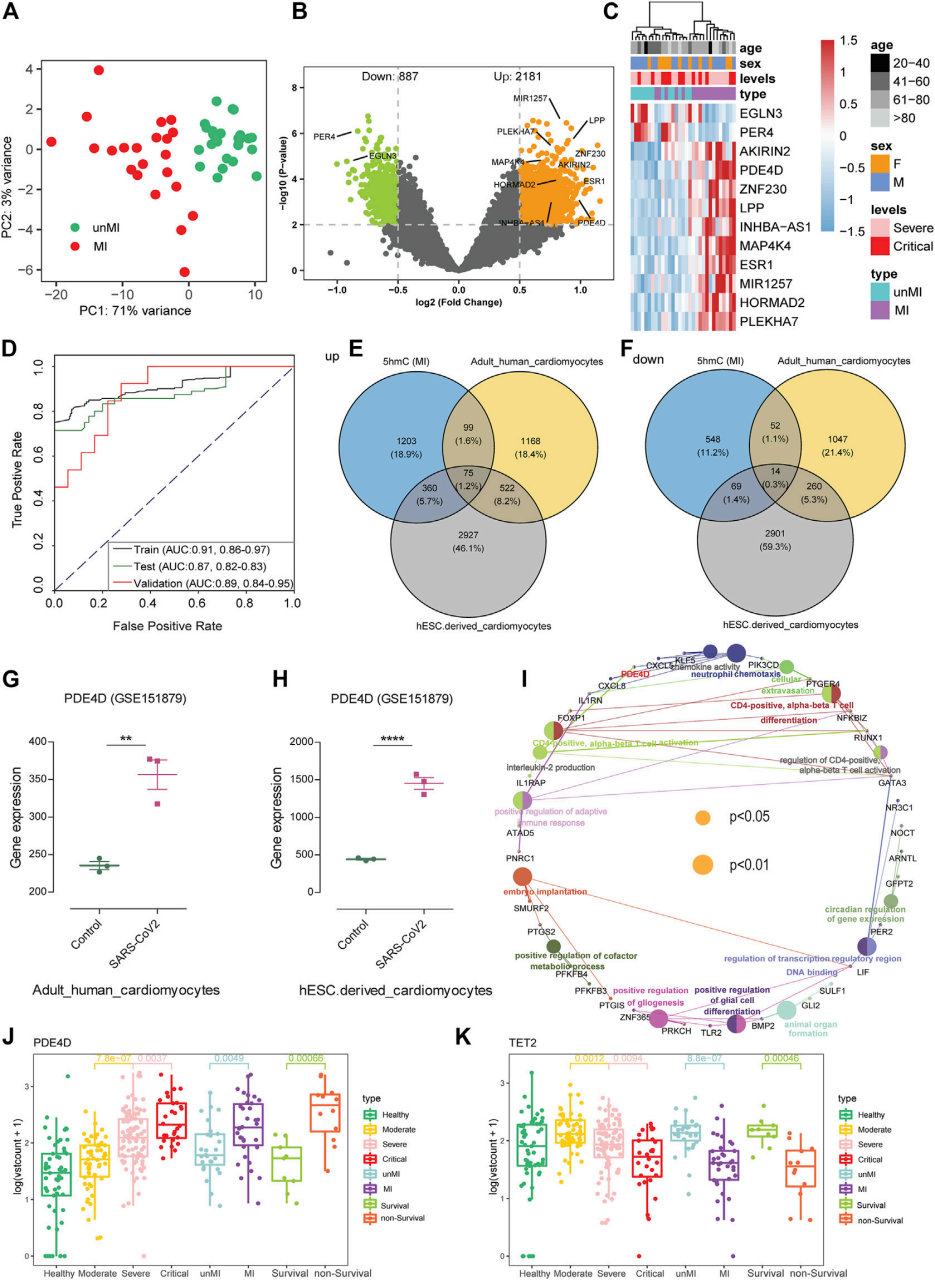
5-Hydroxymethylcytosine signatures as early warning biomarkers for myocardial injury. **(A)** Principal component analysis (PCA) using normalized read counts from patients with myocardial injury (MI) and those without MI (unMI). **(B)** Volcano plot of hMRs (MI patients vs. unMI patients). **(C)** Heatmaps of 12 5hmC markers with myocardial injury patients, levels, sex, and age information labeled in the validation cohort. Hierarchical clustering was performed across DhMRs-associated genes and samples. **(D)** ROC curve of the warning model with DhMRs in training and validation cohorts for myocardial injury. **(E,F)** Venn diagram indicating overlap and specificity of increase **(E)** or decrease **(F)** in myocardial injury patients from 5hmC-seq and RNA-seq (GSE151879) dataset. **(G,H)** The expression data are from the GSE151879 dataset. Each dot represents one healthy person or myocardial injury patients; error bars represent mean values. Statistically significant *p* values are indicated with asterisks (**p < 0.01 and ****p < 0.0001, by *t*-test). **(I)** GO enrichment and Gene-Concept Network with overlapping genes in myocardial injury patients. The node size is proportional to the *p*-value calculated from the network (*p* < 0.05 and *p* < 0.01). **(J,K)** Boxplots of *PDE4D* and *TET2* grouped by healthy people, patients with COVID-19, myocardial injury (MI), and death. Log2 transformation of TMM normalized 5hmC enrichment values were plotted, and Wilcoxon *t*-test was used. 5hmC, 5-hydroxymethylcytosine; hMRs, 5hMc-enriched regions; DhMRs, differentially 5hMc-enriched regions; PDE4D, phosphodiesterase 4D; TET2, ten-eleven translocation 2.

In our studies, about 19.7% (40 cases) of patients with COVID-19 had MI who required ICU admission and ended up with a higher mortality rate than those without MI (Supplementary Figure S4A). Although the pathogenesis and biomarkers of COVID-19 have been reported, few studies addressed complications of COVID-19, especially MI. A total of 40 MI patients and 40 patients without MI were randomly selected from severe and critical patients and utilized in the warning model. Eighty patients were randomly divided into training (*n* = 49) and validation cohorts (*n* = 31). We observed MI patients separated from the without MI patients based on the DhMRs (Figure 4A). Meanwhile, we identified the 5hmC-enriched peaks in MI patients and found that MI patients enrich less peaks than without MI patients in different genomic characteristic regions, such as promoters and exon (Supplementary Figure S4B). Similarly, we identified 3,068 DhMRs (Figure 4B) from the training set and generated a warning model using 12 DhMRs (Supplementary Table S11) from all DhMRs (|log2FoldChange| ≥0.5, *p*-value < 0.01, Supplementary Table S5) was able to effectively differentiate MI patients in the training cohort (Supplementary Figure S4C) and the validation cohort (Figure 4C). In the validation group, the AUC value of this model for early warning the patients whom potentially at risk of MI was 0.89 (95% CI: 0.84 to 0.95, Figure 4D). This indicates that DhMRs could be early warning signs for complications of COVID-19. Besides that, we found that the 5hmC characteristics from cfDNA could be separated from other complications, such as GS and SIC (Supplementary Figures S4D and S4E, Supplementary Table S6 and S7), and separate between the non-survival patients and survival patients (Supplementary Figure S4F, Supplementary Table S8). These results suggest that the 5hmC is a potential tool for the warning of COVID-19 progression and its complications.

### Potential Associations Between 5hmC Markers and Myocardial Injury in patients with COVID-19

To explore the correlation of DhMRs and tissue-specific genes, we initially evaluated the tissue-specific transcriptome profiles of heart tissue from autopsies of healthy and patients with COVID-19, and human embryonic stem cell (hESC)–derived cardiomyocytes in GSE151879 datasets. Comparison between our 5hmC-seq and RNA-seq data, we found that, in addition to the unique genes between three groups, there are 75 and 14 overlapping genes in upregulated genes and downregulated genes, respectively (Figures 4E,F). In particular, we found a common gene, PDE4D, in all three warning models, and PDE4D are highly expressed in the heart tissue from patients with COVID-19, especially in hESC-derived cardiomyocytes (Figures 4G,H). Recent studies showed that uncontrolled inflammation contributes to the disease severity (Del Valle et al., 2020). Our study found the 5hmC modified genes of critical patients enriched in neutrophil-mediated immunity pathways consistent with previous research indicating that the neutrophil elastase inhibitor (Sivelestat) is a promising therapeutic option in COVID-19 with ARDS (Sahebnasagh et al., 2020). To investigate whether there are other targets for the MI or patients with a high risk of death, we examined the pathway enrichment in 75 overlapping genes. We found several immune-related signaling pathways, including chemokine activity, neutrophil chemotaxis, and CD4-positive, alpha-beta T cell activation pathways (Figure 4I). Among them, PDE4D plays an important role in the immune signaling pathway and influences the immune system by activating chemokines and mediating neutrophil chemotaxis (Figure 4I). Interestingly, we found that the 5hmC modification level of PDE4D, a drug target for chronic obstructive pulmonary disease (COPD) (Yuan et al., 2016), was significantly increased in the death group and MI group (Figure 4J). In addition, hydroxymethylation levels were significantly reduced in MI patients (Supplementary figure S4B), and the TET2 enzyme, which can catalyze the conversion of 5-methylcytosine (5mC) to 5hmC, had a lower 5hmC level in the death group and MI patients’ group (Figure 4K).

## Discussion

Recent studies have reported that 5hmC plays a critical role in gene expression regulation and is also a novel tool to identify biomarkers for disease diagnosis and prognosis (Cui et al., 2020). In this study, we profiled genome-wide 5hmC in cfDNA from blood plasma, investigated its association with COVID-19 disease progression, and identified the prognostic factors associated with disease progression, MI, and mortality risk. Our primary analysis found the patients’ groups enrich more peaks than the healthy group, and 5hmC marker genes differed by clinical characteristics of patients at diagnosis. We have identified COVID-19–associated 5hmC signature peaks enriched in the gene bodies and promoter regions (Figure 2A). The COVID-19–associated 5hmC signature gene was enriched in the neutrophil migration pathway, consistent with the previous studies that reported neutrophils and neutrophil extracellular traps drive necroinflammation in COVID-19 (Tomar et al., 2020).

Moreover, we developed a machine learning model based on 5hmC data from patients with COVID-19 at different disease severity classes (moderate, severe, and critical) to warn the disease progression. The 5hmC indicators model improved accuracy compared to the clinical markers such as LDH, IL-6, D-dimer, and NLR (Figures 3B,C,H,I). Overall, these findings suggest the profiled genome-wide 5hmC in cfDNA from blood plasma can be regarded as an early warning of critical illness in COVID-19. Immune phenotyping based on the LDH, IL-6, D-dimer, and NLR is a well-established marker in predicting disease severity and ICU-mortality outcomes in patients with COVID-19 (Yan et al., 2020). However, few studies were focused on the complications, diagnosis, and warning. COVID-19 is regarded as a systemic disease involving multiple systems, including cardiovascular, respiratory, gastrointestinal, and immune system (Iddir et al., 2020; Liu et al., 2020). Our results confirmed that patients who suffer from MI had higher mortality (Supplementary figure S4A), and 5hmC was a potential warning biomarkers of occurring MI in the COVID-19 (Figure 4C).

Our research tried to expand the application of 5hmC markers in the disease, especially for exploring the potential therapeutic targets (Supplementary figure S3E). There are two primary reasons that we think this strategy is reliable. First, Cui et al. performed the 5hmC-Seal and RNA-seq in 19 human tissues derived from 10 organ systems and found that gene-level 5hmC modifications can reflect the gene expression status in different human tissues (Cui et al., 2020). This indicates that 5hmC level is associated with gene expression, consistent with previous studies. Second, several targets used for COVID-19 treatment were also found in our results, including TET2 (Zhang et al., 2021) and neutrophil-mediated immunity pathway (Sahebnasagh et al., 2020). In addition, for the MI, the 12 5hmC-enriched regions (hMRs) were able to differentiate and predict effectively. Interestingly, in a previous study, MAP4K4 (from the 12hMRs) has been to promote cardiac muscle cell death (Virbasius and Czech, 2016). Moreover, MAP4K4 carried a higher 5hmC modification, which positively regulated the gene transcription. MAP4K4 is a key kinase in the mating pathway and is involved in many aspects of cell functions and pathological processes (Yue et al., 2014; Gao et al., 2016). Several studies found MAP4K4 as a therapeutic target in cancer (Gao et al., 2016). Thus, whether the MAP4K4 is a potential therapeutic target for MI needs further study.

We found a potential target for the COVID-19 besides the known targets, such as the PDE4D. In our study, PDE4D was one of the 10 DhMRs that warn severe to critical illness and may mediate cell chemotaxis signaling pathways to affect the neutrophil-related immune system (Supplementary figure S3E). We speculated that the PDE4D could be a potential drug therapeutic target of COVID-19, especially for critical patients. GSE151879 data showed that PDE4D was highly expressed in the heart tissues of patients with COVID-19, especially in hESC-derived cardiomyocytes. It also implicated that PDE4D might play an important role in the immune system, for influencing the immune system by activating chemokines and mediating neutrophil chemotaxis (Figure 4I). In addition, TET2 had a lower 5hmC level in the death group and MI patients’ group (Figure 4K), and vitamin C restoring TET2 function could provide therapy for patients with COVID-19. Recent studies consistently demonstrated that a high dose of intravenous vitamin C could improve outcomes and reduce mortality for patients with COVID-19 (Zhang et al., 2021). We believe that the combination of PDE4D inhibitor and vitamin C is a potential drug combination for the treatment of COVID-19, especially in severely ill patients.

This cohort study has several limitations. First, the number of cases was small, and small cases were used in the machine learning model generation, but our study was the first study using 5hmC as warning biomarkers in patients with COVID-19 and the exploratory study found the relevant targets may have some far-reaching significance. Second, although some clinical information is missing, it does not involve important variables. For example, we used the TnI instead of the electrocardiogram and/or echocardiography for the MI diagnosis. Third, all of the targets should be confirmed by further validation. In current pandemic, we believe that showing this important discovery in advance maybe attract more attention to understanding this new disease. Of course, we are already working on validation studies.

## Conclusion

In conclusion, we identified potential 5hmC markers for patients with COVID-19. This is the first study using 5hmC as early warning biomarkers in patients with COVID-19, and we showed that 5hmC has advanced advantages for COVID-19 progression and MI warning.

## Data Availability

The datasets presented in this study can be found in online repositories. The names of the repository/repositories and accession number(s) can be found in the article/Supplementary Material.

## Abbreviations

5hmC, 5-hydroxymethylcytosine; ARDS, acute respiratory distress syndrome; AUC, area under the curve; CD14, CD14 molecule; COPD, chronic obstructive pulmonary disease; COVID-19, coronavirus disease 2019; CRP C-reactive protein; CVA, cerebrovascular accident; DhMRs, differentially 5hMc-enriched regions; GS, gastrointestinal injury; hMRs, 5hMc-enriched Regions; MAP4K4, mitogen-activated protein kinase kinase kinase kinase 4; MI, myocardial injury; NLR, neutrophil-to-lymphocyte ratio; PDE4D, Phosphodiesterase 4D; RFECV, recursive feature elimination algorithm; SARS-CoV-2, severe acute respiratory syndrome coronavirus 2; SIC, sepsis-induced coagulopathy; TET, ten-eleven translocation; WHO, World Health Organization.
