# Supplementary material for: 5-Hydroxymethylcytosine Signatures in Circulating Cell-Free DNA as Early Warning Biomarkers for COVID-19 Progression and Myocardial Injury
Source: Front Cell Dev Biol. 2022 Jan 6;9:781267. doi: 10.3389/fcell.2021.781267 (PMC8770986; doi:10.3389/fcell.2021.781267)
Supplement: Supplementary file 8 [file Table9.DOCX]

**5-Hydroxymethylcytosine signatures in circulating cell-free DNA as predictive biomarkers for COVID-19 progression and myocardial injury**

Hang-yu Chen ^1*^, Xiao-xiao Li ^2b, 8*^, Chao Li ^2a*^, Hai-chuan Zhu ^4*^, Hong-yan Hou ^5^, Bo Zhang ^5^, Li-ming Cheng ^5^, Hui Hu ^6^, Zhong-xin Lu ^6^, Jia-xing Liu ^4^, Ze-ruo Yang ^8^, Lei Zhang ^8^, Nuo Xu ^8^, Long Chen ^1^, Chuan He ^9^, Chao-ran Dong ^3†^, Qing-gang Ge ^2a†^, Jian Lin ^1†^

**Supplementary Table 9. Coefficients for 15 DhMRs in the logistic regression model trained by the training cohort**

|  | Coefficients | SE | Z | P>\|Z\| | 97.5% CI | GeneID |
| --- | --- | --- | --- | --- | --- | --- |
| Intercept | -13.62 | 3.97 | -3.43 | 0.001 | [-21.40, -5.85] |  |
| chr6_97578573_97578980 | 0.66 | 0.42 | 1.57 | 0.116 | [-0.16, 1.48] | KLHL32 |
| chr19_43866758_43867470 | 0.08 | 0.17 | 0.46 | 0.646 | [-0.26, 0.42] | CD177 |
| chr7_41439865_41440450 | 0.50 | 0.36 | 1.41 | 0.159 | [-0.20, 1.20] | INHBA-AS1 |
| chr3_152485356_152485773 | 0.04 | 0.33 | 0.13 | 0.896 | [-0.60, 0.68] | P2RY1 |
| chr9_21276039_21276628 | 0.45 | 0.33 | 1.38 | 0.169 | [-0.19, 1.09] | IFNA22P |
| chr2_98310400_98311030 | 0.33 | 0.21 | 1.57 | 0.117 | [-0.08, 0.73] | ZAP70 |
| chr5_59517693_59518204 | -0.65 | 0.34 | -1.91 | 0.056 | [-1.31, 0.02] | PDE4D |
| chr1_68715142_68715517 | -0.24 | 0.21 | -1.15 | 0.251 | [-0.66, 0.17] | WLS |
| chr13_72031580_72031950 | 0.24 | 0.36 | 0.67 | 0.506 | [-0.47, 0.95] | DACH1 |
| chr1_12769349_12770105 | -0.39 | 0.15 | -2.58 | 0.010 | [-0.69, -0.09] | AADACL3 |
| chr10_9628645_9629173 | 0.27 | 0.28 | 0.96 | 0.338 | [-0.28, 0.81] | SFTA1P |
| chr5_124121851_124122273 | 1.63 | 0.70 | 2.33 | 0.020 | [ 0.26, 3.00] | ZNF608 |
| chr12_4762930_4763650 | 0.42 | 0.28 | 1.52 | 0.129 | [-0.12, 0.96] | NDUFA9 |
| chr5_55915481_55916120 | 0.81 | 0.42 | 1.95 | 0.052 | [-0.005, 1.63] | MAP3K1 |
| chr6_88004302_88004804 | -0.47 | 0.39 | -1.22 | 0.222 | [-1.23, 0.29] | SMIM8 |

**Abbreviation:** ***DhMRs***, Differentially 5hMc-enriched Regions. ***SE***, standard errors of coefficients; ***z value***, Wald z-statistic value; ***CI***, confidence interval.

**Supplementary Table 10. Coefficients for 10 DhMRs in the logistic regression model trained by the training cohort.**

|  | Coefficients | SE | Z | P>\|Z\| | 97.5% CI | GeneID |
| --- | --- | --- | --- | --- | --- | --- |
| Intercept | 4.10 | 1.05 | 3.89 | 0.000 | [2.03, 6.16] |  |
| chr2_98310458_98310908 | -0.41 | 0.20 | -2.04 | 0.041 | [-0.80，-0.02] | ZAP70 |
| chr3_108275906_108276361 | 0.15 | 0.21 | 0.72 | 0.470 | [-0.27, 0.57] | CIP2A |
| chr3_114931868_114932289 | 0.17 | 0.24 | 0.72 | 0.474 | [-0.30, 0.65] | ZBTB20 |
| chr4_16664143_16664615 | -0.008 | 0.17 | -0.05 | 0.964 | [-0.34, 0.32] | LDB2 |
| chr5_58634218_58634729 | -0.10 | 0.18 | -0.55 | 0.582 | [-0.46, 0.26] | PDE4D |
| chr7_156842323_156843081 | 0.15 | 0.17 | 0.84 | 0.401 | [-0.19, 0.49] | MNX1-AS1 |
| chr8_122427723_122428315 | 0.11 | 0.20 | 0.54 | 0.591 | [-0.29, 0.50] | HAS2-AS1 |
| chr11_128451949_128452270 | -0.18 | 0.14 | -1.28 | 0.201 | [-0.46, 0.10] | ETS1 |
| chr16_84387122_84387490 | -0.18 | 0.14 | -1.31 | 0.191 | [-0.44, 0.09] | ATP2C2 |
| chr20_48218885_48219411 | 0.17 | 0.16 | 1.04 | 0.297 | [-0.15, 0.48] | PTGIS |

**Abbreviation:** ***DhMRs***, Differentially 5hMc-enriched Regions. ***SE***, standard errors of coefficients; ***z value***, Wald z-statistic value; ***CI***, confidence interval.

**Supplementary Table 11. Coefficients for 12 DhMRs in the logistic regression model trained by the training cohort.**

|  | Coefficients | SE | Z | P>\|Z\| | 97.5% CI | GeneID |
| --- | --- | --- | --- | --- | --- | --- |
| Intercept | 2.15 | 3.25 | -0.06 | 0.007 | [-5.68, 4.53] |  |
| chr19_44509716_44510190 | -0.24 | 0.25 | -0.95 | 0.34 | [-0.73, 0.25] | ZNF230 |
| chr14_34575454_34575921 | 0.57 | 0.30 | 1.89 | 0.059 | [-0.02, 1.17] | EGLN3 |
| chr22_30457270_30458221 | 0.002 | 0.11 | 0.02 | 0.985 | [-0.22, 0.22] | HORMAD2 |
| chr7_9403511_9404396 | 0.26 | 0.26 | 0.98 | 0.327 | [-0.26, 0.78] | PER4 |
| chr7_41354441_41355196 | -0.11 | 0.14 | -0.82 | 0.411 | [-0.39, 0.16] | INHBA-AS1 |
| chr3_187971220_187971826 | 0.23 | 0.22 | 1.03 | 0.302 | [-0.21, 0.67] | LPP |
| chr5_59049866_59050599 | 0.13 | 0.10 | 0.22 | 0.043 | [-0.16, 0.35] | PDE4D |
| chr11_16985087_16985664 | -0.08 | 0.17 | -0.48 | 0.629 | [-0.40, 0.24] | PLEKHA7 |
| chr20_60562515_60562912 | -0.08 | 0.17 | -0.45 | 0.653 | [-0.41, 0.26] | MIR1257 |
| Chr6_152238602_152239181 | 0.09 | 0.08 | -0.21 | 0.036 | [0.11, 0.33] | ESR1 |
| chr6_88476279_88476842 | -0.19 | 0.22 | -0.86 | 0.388 | [-0.61, 0.24] | AKIRIN2 |
| chr2_102278044_102278804 | -0.04 | 0.22 | -0.16 | 0.870 | [-0.47, 0.39] | MAP4K4 |

**Abbreviation:** ***DhMRs***, Differentially 5hMc-enriched Regions. ***SE***, standard errors of coefficients; ***z value***, Wald z-statistic value; ***CI***, confidence interval.
